# PET Imaging of Microglial Activation—Beyond Targeting TSPO

**DOI:** 10.3390/molecules23030607

**Published:** 2018-03-08

**Authors:** Bieneke Janssen, Danielle J. Vugts, Albert D. Windhorst, Robert H. Mach

**Affiliations:** 1 Department of Radiology, Perelman School of Medicine, University of Pennsylvania, Philadelphia, PA 19104, USA; bieneke.janssen@pennmedicine.upenn.edu; 2 Department of Radiology & Nuclear Medicine, VU University Medical Center, 1081 HV Amsterdam, The Netherlands; d.vugts@vumc.nl (D.J.V.); ad.windhorst@vumc.nl (A.D.W.)

**Keywords:** positron emission tomography, microglia, neuroinflammation

## Abstract

Neuroinflammation, which involves microglial activation, is thought to play a key role in the development and progression of neurodegenerative diseases and other brain pathologies. Positron emission tomography is an ideal imaging technique for studying biochemical processes in vivo, and particularly for studying the living brain. Neuroinflammation has been traditionally studied using radiotracers targeting the translocator protein 18 kDa, but this comes with certain limitations. The current review describes alternative biological targets that have gained interest for the imaging of microglial activation over recent years, such as the cannabinoid receptor type 2, cyclooxygenase-2, the P2X_7_ receptor and reactive oxygen species, and some promising radiotracers for these targets. Although many advances have been made in the field of neuroinflammation imaging, current radiotracers all target the pro-inflammatory (M1) phenotype of activated microglia, since the number of known biological targets specific for the anti-inflammatory (M2) phenotype that are also suited as a target for radiotracer development is still limited. Next to proceeding the currently available tracers for M1 microglia into the clinic, the development of a suitable radiotracer for M2 microglia would mean a great advance in the field, as this would allow for imaging of the dynamics of microglial activation in different diseases.

## 1. Microglial Activation—Focus on Imaging

**Figure 1:**
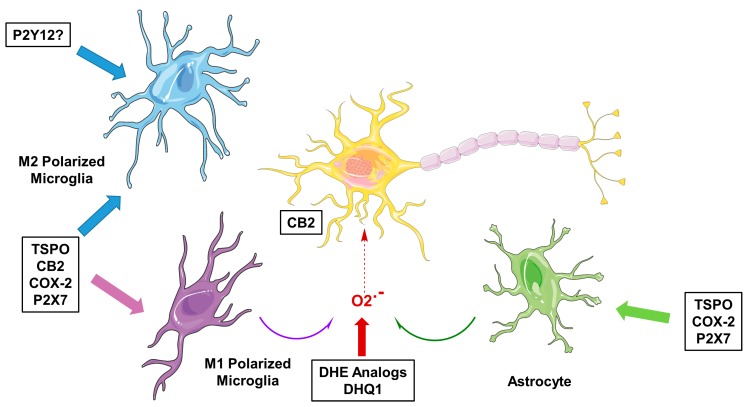
Molecular targets for imaging neuroinflammation in neurodegeneration. TSPO = translocator protein 18 kDa; CB2 = cannabinoid receptor type 2; COX-2 = cyclooxygenase-2; DHE = dihydroethidium; DHQ1 = dihydroquinoline analog.

Microglia are the resident immune cells of the brain and are involved in brain development, maintenance of homeostasis, neuroinflammation and neurodegeneration [1,2]. Microglia are highly dynamic cells, with processes surveilling the brain in homeostasis, while changing morphology to a more amoeboid shape upon activation, for instance when encountering a pathogen or injury to the brain. While altering morphology, cell surface receptor expression and secretion of chemokines and cytokines are altered as well. Although there is a spectrum of activation phenotypes, and transcriptome studies have shown microglial activation to be highly context dependent [2], microglial activation is roughly characterized as being either classic, pro-inflammatory activation (M1) or alternative, anti-inflammatory activation (M2). M1 microglia are recognized to produce pro-inflammatory cytokines such as interleukin (IL)-1β and tumor necrosis factor (TNF)-α and express nicotinamide adenine dinucleotide phosphate (NADPH) oxidase, which generates superoxide and reactive oxygen species (ROS) [2]. On the other hand, M2 microglia promote the healing process, as well as releasing anti-inflammatory factors like IL-10 and tumor growth factor (TGF)-β as well as other growth factors and neurotrophic factors [2]. In vitro, the different phenotypes of activated microglia can be characterized by looking at protein expression using, for example, immunohistochemical staining of post mortem brain samples [3,4,5]. Although a great deal has been learned from these in vitro studies in different neurodegenerative diseases, this has not been translated to an in vivo situation, mainly because of the difficulty of obtaining biopsy specimens from brain. For this purpose, positron emission tomography (PET) would be the ideal medical imaging technique, as it makes use of radioactively labeled molecules that can be specifically directed against a biological target. Traditionally, the target for PET imaging of microglial activation has been the translocator protein 18 kDa (TSPO) [6]. However, tracers targeting TSPO come with certain limitations, mainly due to their high non-specific binding, low brain uptake, and the fact that a TSPO polymorphism causes large differences in binding affinity between subjects [7,8,9]. In addition, TSPO has some drawbacks as a target itself, such as expression in multiple cell types, and upregulation of TSPO has been associated with the M1 activation status of microglia, and, to a lesser extent, with the M2 activation status [10]. Furthermore, it was recently reported that TSPO expression increases by about nine-fold under neuroinflammatory conditions in rodent macrophages and microglia, but in humans, no significant increase was found [11]. Moreover, under pro-inflammatory conditions, a decrease in TSPO protein expression was observed in human adult microglia and monocyte-derived macrophages [11]. Although more investigation, especially in vivo, is needed, these results suggest that increased signals obtained in humans using PET tracers targeting the TSPO do not necessarily reflect increased microglial activation, but merely provide a measure of microglial/macrophage density. For these reasons, more and more effort is being put into the search for new targets and tracers for PET imaging of microglial activation. Specific interest lies in the imaging of the different microglial phenotypes. Apart from alterations in the expression of TSPO, the expression of several other receptors and enzymes is altered during microglial activation (Figure 1). The current review focuses on the recent progress made in the development of PET tracers for neuroinflammation imaging, specifically for the cannabinoid receptor type 2 (CB2), cyclooxygenase-2 (COX-2), purinergic receptor P2X_7_ and ROS.

## 2. PET Tracers Targeting the Cannabinoid Receptor Type 2

**Figure 2:**
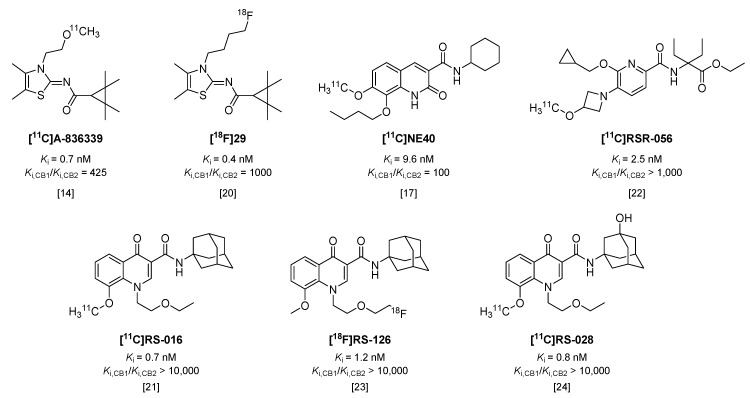
Radiotracers targeting the cannabinoid receptor type 2 (CB2).

Cannabinoid receptors are G-protein coupled receptors, of which two subtypes are known to date. Whereas cannabinoid receptor type 1 (CB1) is constitutively expressed in the central nervous system (CNS), type 2 (CB2) is predominantly expressed in peripheral organs [12]. However, CB2 is also expressed on microglia and neurons [13], and its expression in particularly microglia increases significantly under neuroinflammatory conditions [12,13]. As a consequence, a number of tracers have been recently investigated in animal models of microglial activation. [^11^C]A-836339 (Figure 2, *K*_i_ = 0.7 nM, 425-fold selective over CB1 [14,15]) showed an increase in specific brain uptake in mice, 5 days after systemic injection of lipopolysaccharide (LPS), compared with control mice [14]; however, in a later study with the same tracer, Pottier et al. could not reproduce these results in LPS-treated rats [16]. Two other rat models were included in the latter study (α-amino-3-hydroxy-5-methyl-4-isoxazolepropionic acid (AMPA) and experimental stroke) and neither model demonstrated increased tracer uptake [16]. Likewise, uptake of [^11^C]NE40 (Figure 2, *K*_i_ = 9.6 nM, 100-fold selective over CB1 [17]) was not significantly increased in brains of rats after experimental stroke, compared with sham-operated animals [18]. In contrast to this, more recently, an increased uptake of [^11^C]NE40 was observed one day after experimental stroke in rats, but uptake decreased to baseline levels at later time-points [19]. Using an ^18^F-labeled analog of A-836339, [^18^F]29 (Figure 2, *K*_i_ = 0.4 nM, 1000-fold selective over CB1 [20]**)**, increased uptake was observed in brains of mice treated with LPS, but with only 7% of intact tracer left in plasma at 30 min post injection (p.i.) [20], its rapid metabolic degradation will hamper further application of this tracer.

Slavik et al. were able to show increased uptake in mouse brains after systemic administration of LPS with both [^11^C]RS-016 (Figure 2, *K*_i_ = 0.7 nM, >10,000-fold selectivity over CB1 [21]) and [^11^C]RSR-056 (*K*_i_ = 2.5 nM, >1000-fold selective over CB1 [22]), although uptake of [^11^C]RSR-056 could only be blocked marginally, suggesting a high level of non-specific binding [22]. The same results could not be obtained with analog [^18^F]RS-126 (Figure 2, *K*_i_ = 1.2 nM, >10,000-fold selective over CB1) [23], and in a later in vitro study in post mortem tissue of amyotrophic lateral sclerosis (ALS) patients, a high amount of non-specific binding was observed [24]. Therefore, another ^11^C-labeled analog was synthesized, which did show increased binding to ALS patient tissue compared with control tissue, but in vivo in mice [^11^C]RS-028 (Figure 2, *K*_i_ = 0.8 nM, >10,000 selective over CB1) showed rapid washout of spleen (high abundance of CB2) and low brain uptake, leading authors to conclude this tracer is not optimal for in vivo application [24].

To date, one tracer targeting CB2 has been investigated in humans. [^11^C]NE40 showed favorable fast brain uptake and washout in healthy human subjects [25]. Unfortunately, in a follow-up study in healthy controls and patients with Alzheimer’s disease (AD), decreased brain uptake of [^11^C]NE40 in AD patients compared with healthy control subjects was shown [26], even though upregulated expression of CB2 has been previously demonstrated in post mortem AD brain tissue [27]. The authors concluded that the decrease in uptake in the brain of AD patients is possibly a result of neuronal loss in late stage AD [26], leading to an overall decrease in CB2 expression, even though CB2 may be upregulated in microglia. This is supported by a study in transgenic AD mice using [^11^C]A-836339, in which increased tracer binding was observed in brains of AD mice compared with control animals [13]. However, the transgenic model is not accompanied by neuronal loss, and extensive immunohistochemical staining studies showed that, in control mice, CB2 expression is mainly localized on neurons, whereas in transgenic AD mice, CB2 is mainly expressed in microglia [13]. Importantly, the intensity of the staining and thus, the expression of CB2 in neurons did not differ between control mice and transgenic AD mice, and therefore, increased tracer uptake could be attributed to upregulated CB2 expression in glial cells [13].

In summary, the upregulation of CB2 in neuroinflammation still needs to be further investigated. In particular with respect to neurodegenerative diseases, as in more advanced stages of these diseases, neuronal loss is known to occur. Therefore, CB2 may only be useful in the earliest stages of neurodegenerative diseases. In addition, as the above described studies have shown, high selectivity for CB2 over CB1 of radiotracers is necessary to eliminate a non-specific PET signal due to the high abundance of CB1 in CNS.

## 3. PET Tracers Targeting Cyclooxygenase-2

**Figure 3:**
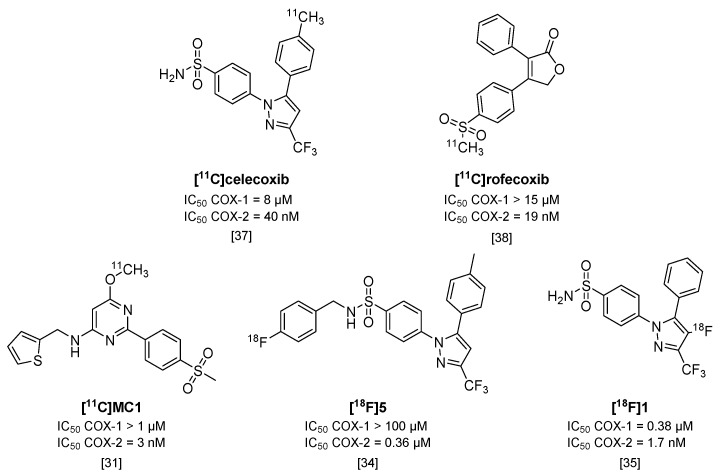
Radiolabeled inhibitors of cyclooxygenase-2 (COX-2).

Cyclooxygenases (COX) are involved in the arachidonic acid cascade and activation of inflammatory pathways, such as Nuclear Factor (NF)-κB, leading to the release of prostaglandins, chemokines, cytokines and ROS [28]. Both isoforms (COX-1 and COX-2) are expressed in the brain, but regulatory functions and cell localization differ between the two. COX-2 has been of main interest as a target in neuroinflammation, as its expression is low in healthy brains but is rapidly overexpressed under inflammatory conditions [28,29]. However, COX-1 has also been implicated as having a role in neuroinflammation [28] and conflicting evidence exists for the expression and upregulation of COX-2 under inflammatory conditions [29]. To investigate the potential of COX-1 or COX-2 as targets for imaging of neuroinflammation, radiotracers should have high selectivity for one isoform over the other. Only a limited amount of isoform-selective tracers have been published, and COX-2 targeting tracers show high blood pool retention and limited uptake in target organs. These tracers also show high amounts of non-specific binding, relatively low affinities (>50 nM) and rapid metabolism or, in the case of ^18^F-labeled compounds, substantial defluorination [29,30]. Some promising COX-2 selective radiotracers are depicted in Figure 3. One of these tracers, [^11^C]MC1 (IC_50_ COX-2 = 3 nM; COX-1 > 1000 nM) [31], was recently used in a LPS-induced neuroinflammation model in rhesus monkeys together with COX-1 selective tracer [^11^C]PS13 (IC_50_ COX-1 = 1 nM; COX-2 > 1000 nM) [32]. Interestingly, this study showed an upregulation of COX-2, but not COX-1, after LPS-induced neuroinflammation in rhesus monkey brains [33].

An ^18^F-labeled analog of celecoxib ([^18^F]**5**; Figure 3) [34] was evaluated in colorectal cancer cells in vitro, but uptake of the tracer could not be blocked with either celecoxib or rofecoxib. Although [^18^F]**5** showed increased selectivity for COX-2 over COX-1 (IC_50_ > 100 µM) compared with celecoxib, its IC_50_ value for COX-2 also decreased to 0.36 µM, which might explain the non-selectivity in blocking experiments. In addition, although [^18^F]**5** was metabolically stable, the tracer was quickly cleared in vivo in COX-2 expressing tumor-bearing mice, which resulted in a lack of uptake in the tumor compared with the uptake observed in vitro, and is therefore not a suitable candidate for COX-2 imaging studies [34]. Another metabolically stable ^18^F-labeled analog of celecoxib, [^18^F]**1** (IC_50_ COX-2 = 1.7 nM; IC_50_ COX-1 = 0.38 µM [35]), was obtained via the non-standard route of electrochemical radiofluorination [36] to attach the fluorine-18 atom to the pyrazole ring (Figure 3). Although this led to low yields (2% radiochemical yield) and low molar activity (~110 MBq/µmol), [^18^F]**1** could be a valuable COX-2 radiotracer in vivo, once, as indicated by the authors, an efficient radiosynthesis method is identified, given its good in vitro affinity, and high metabolic stability in mice (>95% intact tracer at 60 min p.i. in blood), and its high brain uptake (2% ID/g in mouse brain at 60 min p.i.).

To summarize, failure to show COX-2 upregulation in animal models of neuroinflammation and human disease may have been caused by the suboptimal properties of the tracers evaluated to date (i.e., [^11^C]celecoxib [37] and [^11^C]rofecoxib [38]), which includes either poor selectivity for COX-2 over COX-1, or a high level of non-specific binding. Therefore, the newly developed and selective radiotracers mentioned here (e.g., [^11^C]MC1) should be evaluated in human disease, to assess their potential in a clinical setting.

## 4. PET Tracers Targeting the P2X_7_ Receptor

The P2X_7_ receptor (P2X_7_R) is expressed in multiple cell types of the myeloid cell lineage, and although conflicting evidence exists for P2X_7_R expression in astrocytes and neurons, in CNS the receptor is mainly expressed in microglia. The natural agonist of P2X_7_R is adenosine triphosphate (ATP), but as the affinity of ATP for P2X_7_R is low, the receptor is only activated at high (mM) concentrations of ATP. Therefore, the receptor is regarded as silent in normal physiology, but functionally upregulated in case of an imbalance in ATP concentration in pathological conditions [39]. Activation of P2X_7_R is involved in a diverse series of signaling pathways that are linked to neuroinflammation [40] and is the key step in the activation of the inflammasome, leading to the release of pro-inflammatory cytokines like IL-1β [39]. In addition, the generation of ROS following P2X_7_ receptor activation by ATP or BzATP has been described for multiple cell types, including microglia [40,41]. P2X_7_R is therefore associated with the pro-inflammatory phenotype of microglia, and its functional expression is usually upregulated in CNS disease [39], which makes it an interesting target for both drugs and PET tracers.

**Figure 4:**
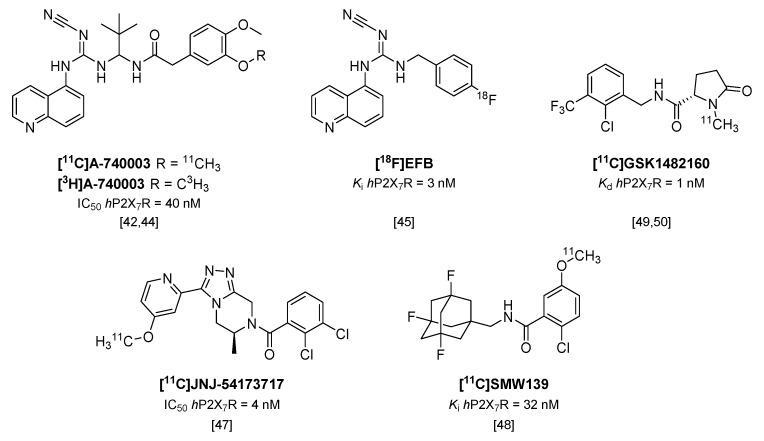
Radiolabeled P2X_7_ receptor antagonists.

Although P2X_7_R antagonist [^11^C]A-740003 (Figure 4) has already been shown to not enter the brain [42], recently, the tritiated analog of this potent P2X_7_R antagonist ([^3^H]A-740003; IC_50_ *h*P2X_7_R = 40 nM; IC_50_ *r*P2X_7_R = 18 nM [43]) was used in an in vitro study in post mortem brain sections of multiple sclerosis (MS) patients and rat brain sections of a rat model of MS (experimental autoimmune encephalomyelitis; EAE) [44]. P2X_7_R was shown to be associated with the pro-inflammatory phenotype of microglia, and was highly expressed in active MS lesions in human brain compared with normal appearing white matter and chronic active lesions. In addition, in brain sections of the EAE rat model, [^3^H]A-740003 binding increased during the peak of the disease (14 days after immunization). In both rat and human brain sections, increased tracer binding was confirmed with immunohistochemical staining for P2X_7_R.

An analog of another cyanoguanidine containing compound (A-804598), was recently labeled with fluorine-18 [45]. [^18^F]EFB (Figure 4) showed good affinity towards both human and rat P2X_7_R (*K*_i_ of 3 and 36 nM, respectively), but low brain uptake was observed in both healthy rats and rats treated with LPS prior to PET scanning [45], and thus the application of [^18^F]EFB in imaging of microglial activation will be limited. 

Two carbon-11 labeled P2X_7_R antagonists of different compound classes (Figure 4), [^11^C]JNJ-54173717 (IC_50_ *h*P2X_7_R = 4 nM) and [^11^C]SMW139 (*K*_i_ *h*P2X_7_R = 32 nM [46]), were evaluated in a humanized rat model, in which the human P2X_7_ receptor was locally expressed in striatum via an adeno-associated viral vector [47,48]. Both tracers entered the rat brain and showed excellent uptake in the *h*P2X_7_R overexpressing striatum (standardized uptake value (SUV) 0.8 and 2.1 at 10 min p.i., respectively) compared with the contralateral striatum (SUV 0.6 and 1.4 at 10 min p.i., respectively). In addition, [^11^C]JNJ-54173717 also showed high initial brain uptake (SUV 3.3) in non-human primates [47], which likely enables translation to humans, and [^11^C]JNJ-54173717 is expected to proceed to clinical evaluation. Although P2X_7_R overexpression in post mortem brain material of AD patients could not be shown using [^11^C]SMW139 [48], a clinical study with this tracer is currently ongoing in patients diagnosed with MS, based on the findings of P2X_7_R upregulation in active MS lesions described by Beaino et al. [44].

[^11^C]GSK1482160 (K_d_ *h*P2X_7_R = 1 nM/*K*_i_ *h*P2X_7_R = 3 nM) was recently evaluated in a mouse model of LPS-induced neuroinflammation [49] and the EAE rat model [50]. In contrast to other studies using the LPS model of neuroinflammation [45,51] in which expression levels of Iba1 and P2X_7_R were found to peak as early as 12 h post injection of LPS, the study by Territo et al. showed highest expression of Iba1 only at 72 h p.i. Biodistribution studies in LPS-treated (5 mg/kg i.p.) and saline-treated control mice revealed an increased uptake of [^11^C]GSK1482160 in LPS-treated mice compared with saline-treated mice in all organs studied (2.9–5.7-fold) [49]. Small animal PET imaging revealed a stable uptake in tissue within 10 minutes after tracer injection, and increased uptake in the brains of LPS-treated mice (3.6-fold) was confirmed. In another study, [^11^C]GSK1482160 was shown to enter the brains of rhesus macaques with an SUV maximum (2.7) at around 70 min p.i. [50]. The same group showed increased tracer binding in the lumbar spinal cord at the peak of the disease in EAE rats (12–14 days post immunization) compared with healthy rats in an autoradiography study, but failed to show this increase in vivo due to a stated insufficient affinity of [^11^C]GSK1482160 for the rat P2X_7_R [50].

Over the last few years, P2X_7_R has gained interest as a target for PET imaging of microglial activation and may be a promising alternative for targeting TSPO. In addition, P2X_7_R may even be more useful as a target for imaging of microglial activation, as it has been shown to be associated predominantly with the pro-inflammatory microglial phenotype [44,45,49]. In in vitro situations, in both animal models of disease and human tissue sections, P2X_7_R tracers have shown promise in the ability to show the presence of neuroinflammation. However, the promising preclinical findings still need to be confirmed in a human situation, as P2X_7_R overactivation may differ between diseases and possibly even disease stages. In addition, no data are available on the actual expression levels of P2X_7_R in humans, or differences therein in health and disease. To uncover the potential of targeting P2X_7_R for PET imaging of microglial activation, several P2X_7_R tracers are currently being evaluated in clinical trials.

## 5. PET Radiotracers for Imaging ROS

Oxidative stress results from the formation of pro-inflammatory microglia and astrocyte activation, which are sources of nitric oxide (NO) and superoxide due to their increased expression of iNOS and high levels of NADPH oxidase activity (Figure 1). Superoxide is normally removed from cells by the action of superoxide dismutase (SOD). However, under high levels of NADPH oxidase activity, superoxide can overwhelm these “protective mechanisms” and react with NO to form peroxynitrite (ONOO-). Peroxynitrite is a highly reactive oxidant which can damage macromolecules within the cytoplasm and nucleus, including DNA strand breaks, lipid peroxidation and the oxidation of sulfur groups in proteins. The formation of DNA strand breaks is thought to lead to the activation of cell death pathways via caspase-mediated or noncaspase-mediated mechanisms (i.e., necroptosis or parthanatos) [52]. The development of PET radiotracers capable of imaging superoxide levels in the CNS is expected to provide a sensitive means for imaging pro-inflammatory neuroinflammation in neurodegenerative disorders such as AD [52]. In addition, the recognition of the role of NADPH oxidase as a key mediator of oxidative stress via its production of superoxide has led to the development of inhibitors of this enzyme as therapeutic targets for neurodegenerative diseases. Therefore, the availability of a PET radiotracer for imaging superoxide levels in the CNS is expected to serve as a sensitive measure of the therapeutic efficacy of a putative NADPH oxidase inhibitor. 

**Figure 5:**
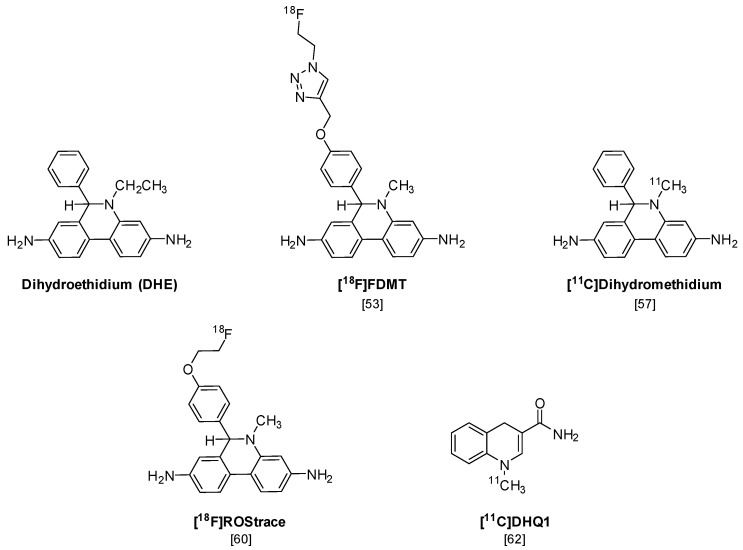
Structures of the positron emission tomography (PET) radiotracers for imaging increased levels of reactive oxygen species (ROS) in neuroinflammation.

The first PET radiotracer for imaging superoxide is [^18^F]FDMT (Figure 5), an ^18^F-labeled analog of the fluorescent probe dihydroethidium (DHE), that was synthesized using “click” chemistry [53]. DHE has been previously shown to provide a sensitive measure of superoxide levels in cells and tissues, using microscopy and optical imaging techniques [54,55]. Although [^18^F]FDMT showed promising results in an animal model of adriamycin-induced model of cardiotoxicity, this radiotracer did not cross the blood-brain barrier (BBB) and is not capable of imaging the increased levels of superoxide that occur during neuroinflammation. 

A number of radiolabeled analogs of dihydromethidium (a.k.a. hydromethidine), which is the corresponding *N*-methyl analog of DHE, have been synthesized and evaluated in vitro and in vivo [56,57]. [^3^H]Dihydromethidium is oxidized by both superoxide and hydroxyl radicals and has been evaluated in animal models of stroke and cisplatin-induced nephrotoxicity [58,59]. A recent paper has also reported the radiosynthesis and preliminary in vivo evaluation of [^11^C]dihydromethidium [57]. This compound shows high brain uptake in microPET imaging studies, but a detailed analysis of the mechanisms of uptake and trapping of this radiotracer was not conducted.

**Figure 6:**
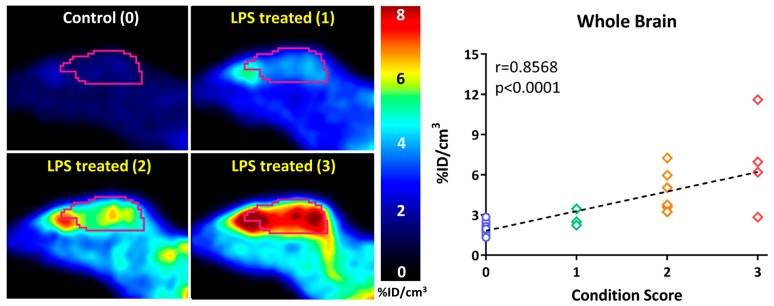
The uptake of [^18^F]ROStrace correlates with the condition score (degree of “sickness”) following treatment with lipopolysaccharide (LPS).

**Figure 7:**
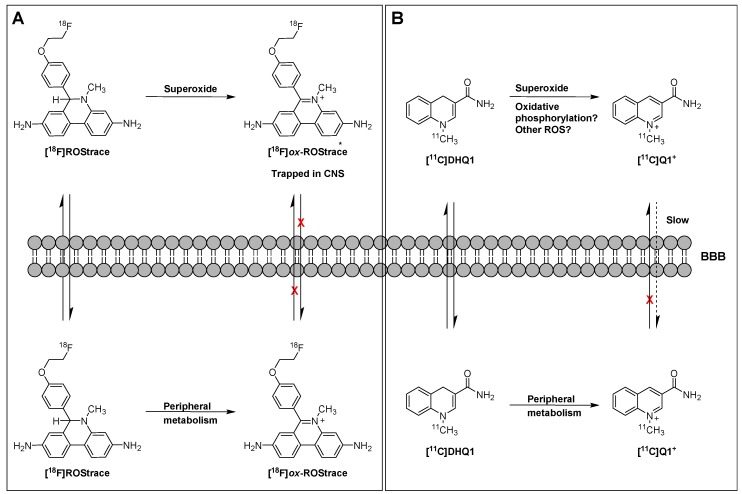
Mechanism of trapping of [^18^F]ROStrace (**A**) and [^11^C]DHQ1 (**B**).

In a follow-up study to their earlier work with [^18^F]FDMT, Mach and coworkers reported the synthesis, in vitro characterization, and in vivo evaluation of [^18^F]ROStrace in an LPS-induced mouse model of neuroinflammation [60]. In this study, the investigators reported that replacing the corresponding triazole “click” moiety with the traditional [^18^F]2-fluoroethoxy group resulted in a compound that freely crossed the BBB. A key observation in this study was the high variability in uptake of [^18^F]ROStrace in the LPS-treated animals. However, when the investigators compared the uptake of [^18^F]ROStrace with the degree of “sickness” following the LPS-treatment by using the scoring criteria outlined by Carstens and Moberg [61] for recognizing pain and distress in laboratory animals, there was a high correlation between radiotracer uptake and degree of sickness induced by LPS (Figure 6) [60]. The authors also conducted a detailed metabolite analysis study, confirming that the radioactive species in the LPS-treated animals was primarily the oxidized form of [^18^F]ROStrace, [^18^F]*ox*-ROStrace. Since [^18^F]*ox*-ROStrace does not cross the BBB, these results confirm the mechanism of uptake and trapping outlined in Figure 7A. Since ROStrace is oxidized by superoxide and not hydrogen peroxide and the hydroxyl radical, this trapping occurs via the oxidation of [^18^F]ROStrace to [^18^F]*ox*-ROStrace by superoxide.

A second compound that has shown promise in imaging oxidative stress is the dihydroquinoline analog, [^11^C]DHQ1 (Figure 5) [62]. [^11^C]DHQ1 is an analog of NADH/NADPH and has been used previously as a redox carrier in the delivery of drugs to the CNS. It is capable of crossing the BBB and, like the DHE analogs described above, is trapped in the brain by oxidation to a charged species (Figure 7B). Since pretreatment with the NOX2 inhibitor, apocyanin, results in a reduction in uptake of [^11^C]DHQ1, part of its trapping mechanism can be attributed to the oxidation of the dihydro species to the corresponding *N*-methylquinolinium species. However, this compound is also thought to be a substrate for enzymes for the oxidation of NADH/NADPH in oxidative phosphorylation, so its trapping mechanism is not limited to the presence of elevated levels of superoxide. Furthermore, no information was provided on its relative reactivity to other oxidizing species, such as hydrogen peroxide (H_2_O_2_) or hypochlorous acid (HOCl). Therefore, this tracer is best described as providing a nonselective measure of oxidative stress. 

## 6. Concluding Remarks and Future Directions

Whereas traditionally, the development of new PET tracers for imaging of neuroinflammation has been largely focused on improving the available ligands for TSPO, in recent years, attention has begun to shift towards the development of radiotracers for alternative targets. Since the activation of microglia is highly dynamic, and protein expression is dependent on both microglial phenotype and microglial environment, (over)expression of a certain target protein may differ per disease, or even be dependent on disease stage. Therefore, being able to visualize multiple targets in the living brain is of utmost importance to gain more insight in this dynamic process. This is especially true for the human situation, given the difficulty of obtaining biopsy specimens of brain tissue. As discussed in the current review, several promising new radiotracers have been developed, targeting CB2, COX-2, P2X_7_R and ROS. Although all of these targets are involved in the pro-inflammatory phenotype (M1) of microglia, it may very well be that not every tracer/target is equally suitable for imaging microglial activation in every disease, due to the aforementioned dynamics in protein expression in microglial phenotypes. In addition, while radiotracers are often evaluated in rodent models of excitotoxin-induced neuroinflammation or models of human disease, these are not necessarily representative of human disease, as was recently also reported for TSPO [11]. To evaluate the potential of the biological targets discussed in this review as valuable targets for PET imaging, it will be very interesting to see the outcome of the clinical studies that are currently ongoing for several tracers. Nevertheless, all of these targets are specific for the pro-inflammatory phenotype (M1) of activated microglia, and, to get a complete view of the neuroinflammatory process, it is important to focus on the anti-inflammatory phenotype (M2) as well. However, tracer development is limited by the availability of targetable biomolecules specific for this phenotype. One target of high interest is the P2Y_12_ receptor (P2Y_12_R), a G-protein coupled receptor that is highly overexpressed in the anti-inflammatory phenotype compared with the pro-inflammatory and resting state phenotypes [2,63,64]. Moreover, the expression of P2Y_12_R in CNS is limited to microglia only [2] and could therefore exclude any PET signals from infiltrating monocytes and macrophages. Two recent autoradiography studies using a carbon-11 labeled P2Y_12_R antagonist on brain sections of the EAE rat model and MS patients [44] and rodent stroke models and a patient deceased from stroke [65] demonstrated the possibility of visualizing the anti-inflammatory subset of microglia cells (i.e., M2-polarized microglia). Unfortunately, despite the fact that many potent P2Y_12_R antagonists have been developed due to their use as anti-coagulants, a (radiolabeled) compound that crosses the BBB is still lacking. The development of a brain-penetrant tracer that targets the anti-inflammatory phenotype of activated microglia would initiate a great advance in the field of neuroinflammation imaging, as a combined tracer study (pro- vs. anti-inflammatory) could provide new insights into the dynamics of microglial activation in health and disease.
